# Supplementary material for: Development and preclinical evaluation of a cable-clamp fixation device for a disrupted pubic symphysis
Source: Commun Med (Lond). 2022 Dec 22;2:164. doi: 10.1038/s43856-022-00227-z (PMC9780275; doi:10.1038/s43856-022-00227-z)
Supplement: Supplementary file 1 — Description of Additional Supplementary Files [file 43856_2022_227_MOESM1_ESM.pdf]

Description of Additional Supplementary Files

Supplementary Video 1. Trans-obturator surgical approach through a horizontal incision in a male cadaver, involving preparation of the pubic symphysis, opening of the obturator foramen on both sites, placement of the CCAO implant, screw fixation, cable threading within the guiding structure, cable tensioning to re-join the pubis symphysis, securement of the cable, and wound closure.

Supplementary Video 2. Trans-obturator surgical approach in a female cadaver, involving placement of the CCAP implant, encirclement of the pubic symphysis with a steel cable using a cable passer, threading of the cable into the guiding structure of the clamp, tensioning and securing of the implant. Here, visual access in the absence of symphyseal gapping or an additional cranial incision was very limited. The risk of bladder laceration using this technique is shown by slitting the bladder with the cable passer in one of the specimens.

Supplementary Video 3. Trans-obturator approach through a midline incision to the pubic symphysis, involving preparation of the cranio-medial border of the obturator foramen, insertion of the first and second CCAO implants with the cable already threaded through the guiding channels, tensioning and securement of the cable cerclage using commercially available instruments, screw fixation of the clamp to avoid dislocation or loosening, and wound closure.

Supplementary Video 4. Trans-obturator approach through a midline incision, involving preparation of the cranio-medial border of the obturator foramen, cranial extension into the abdominal wall, palpation of the obturator membrane, careful passing of the cable through the obturator foramen, placement of the CCAP with the cable inside the pelvis, rejoining of the pubic symphysis, securement of the cable, and closure of the abdominal wall, subcutaneous tissue, and skin.

Supplementary Data 1. Raw data of our biomechanical analysis including the material testing machine and the optical measuring system. Data includes synthetic bone and cadaver specimens.
