# Supplementary material for: Development and preclinical evaluation of a cable-clamp fixation device for a disrupted pubic symphysis
Source: Commun Med (Lond). 2022 Dec 22;2:164. doi: 10.1038/s43856-022-00227-z (PMC9780275; doi:10.1038/s43856-022-00227-z)
Supplement: Supplementary file 2 — Supplementary Information [file 43856_2022_227_MOESM2_ESM.pdf]

Supplementary Information

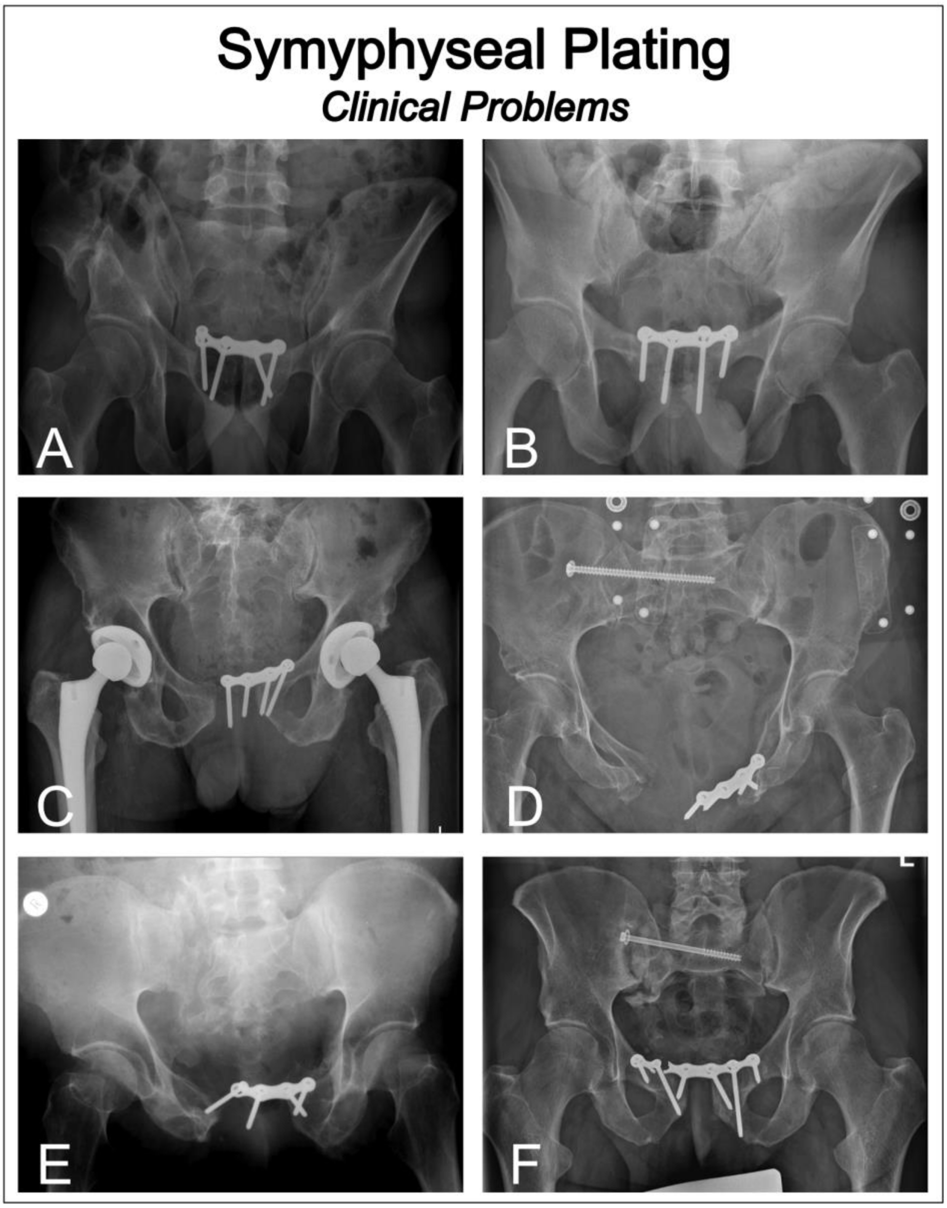

Supplementary Figure 1.

Title: X-ray images of common clinical problems encountered by patients with a symphyseal plate (SP), demonstrating the need for alternative fixation techniques.

Legend: (A) Screw loosening that does not require surgical revision. **(B)** Implant failure caused by screw breakage. Complete screw detachment and symphyseal re-gapping usually requires revision surgery and can be caused by **(C)** poor bone quality, **(D)** fracture of the pubic ramus, or **(E)** instability of the posterior pelvic ring. **(F)** Implant failure caused by plate breakage.

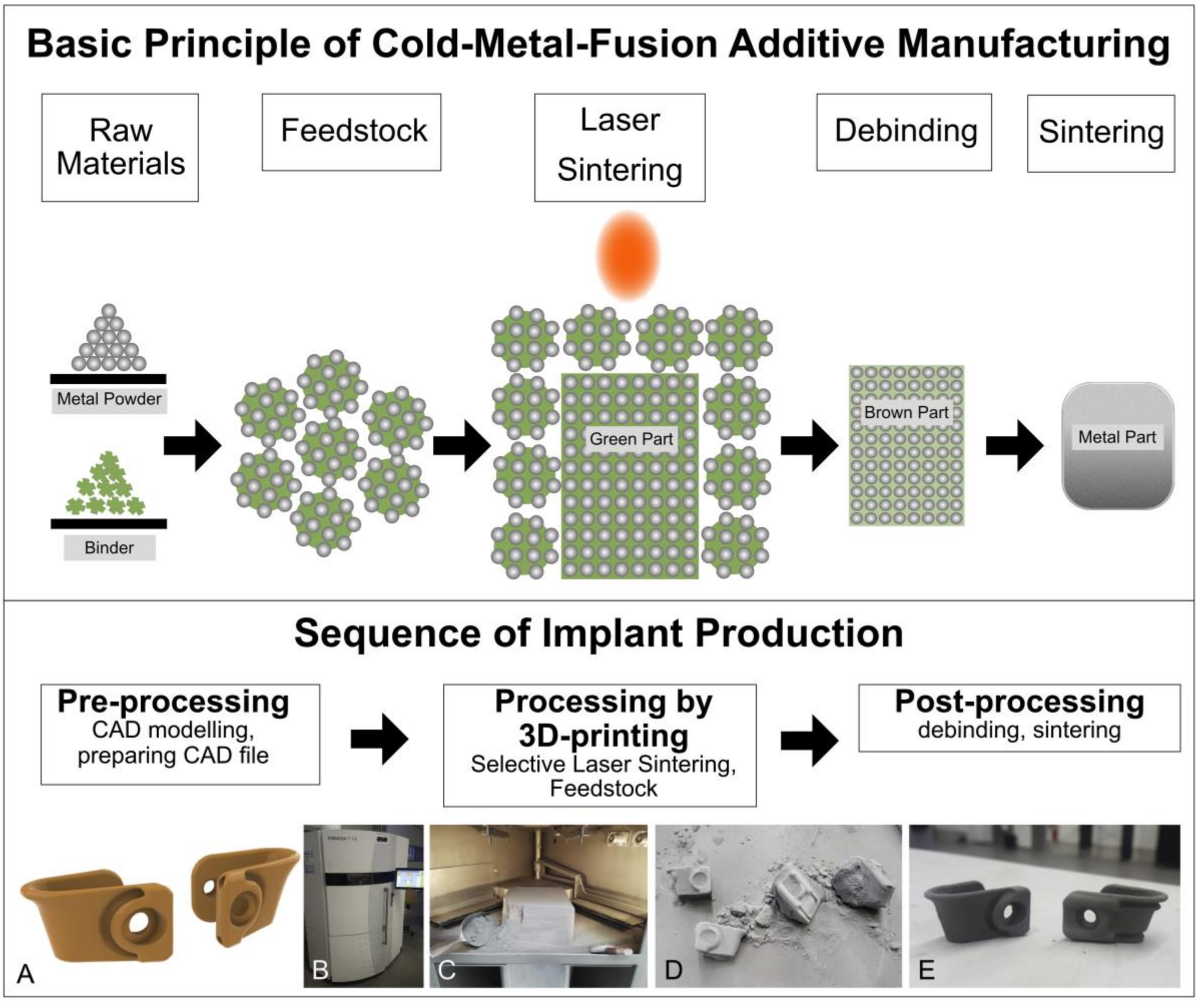

Supplementary Figure 2.

Title: Basic principle of three-dimensional (3D) printing using cold metal fusion.

Legend: A mixture of titanium and synthetic powder is used as the feedstock in the selective laser sintering process, yielding a green part. During debinding, most of the binder is dissolved, yielding a brown part. Finally, sintering extracts the remaining binder, leaving a solid metal part. The manufacture of a complete metallic orthopedic implant involved **(A)** computer-aided design (CAD), **(B,C)** 3D printing, **(D)** debinding, and **(E)** sintering.

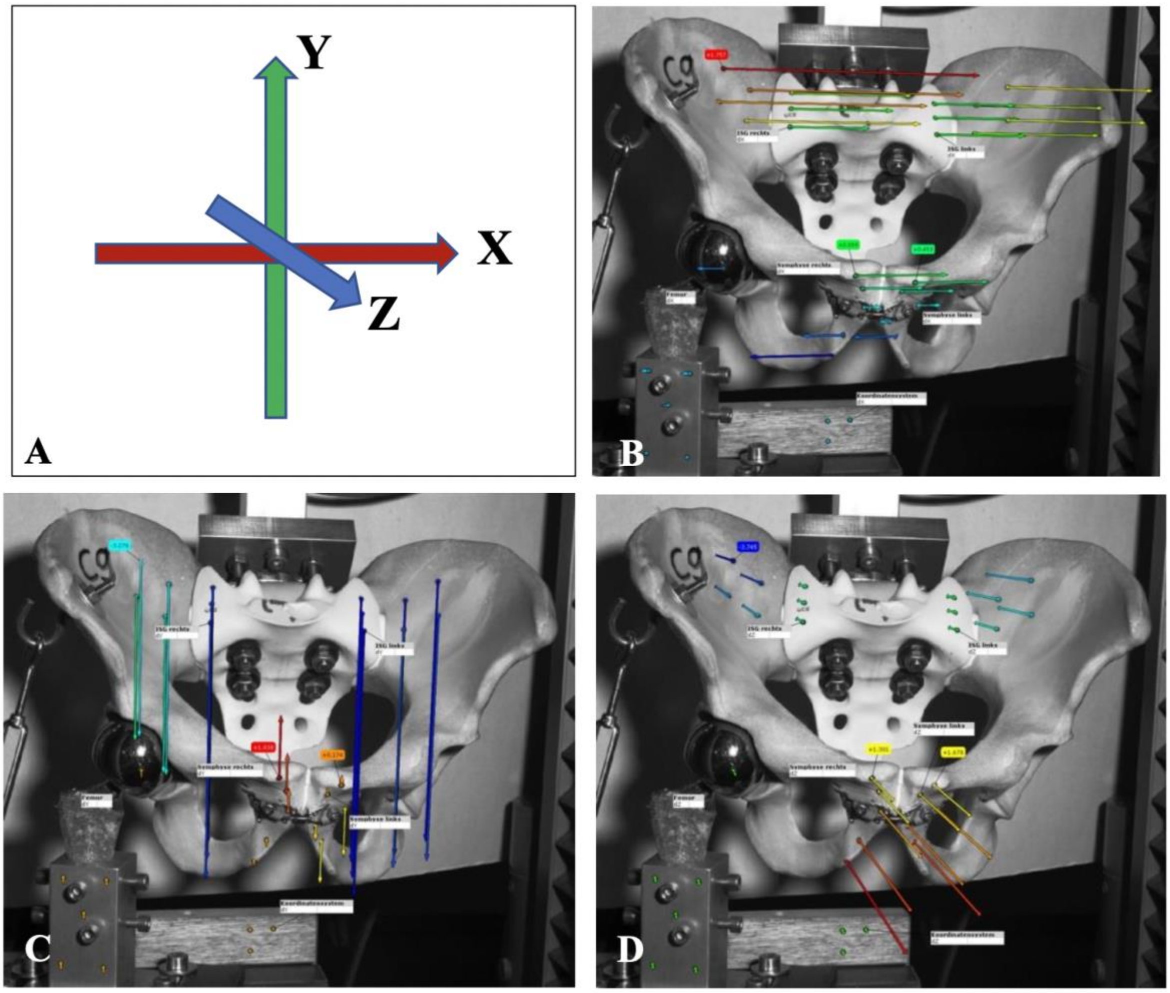

Supplementary Figure 3.

Title: The 3D-motion capturing.

Legend: (A) The system was calibrated and movement measured in the x-, y-, and z-axis. (B) Horizontal movement of the x-axis was less important in our single leg stance test because there was no tension at the pubic symphysis. (C) Vertical movement along the y-axis was the most reliable parameter for our testing because it documented the vertical sheer stress. (D) Movement in the anterior-posterior direction along the z-axis was documented but less relevant.
